# Calcium Signaling Pathway Is Associated with the Long-Term Clinical Response to Selective Serotonin Reuptake Inhibitors (SSRI) and SSRI with Antipsychotics in Patients with Obsessive-Compulsive Disorder

**DOI:** 10.1371/journal.pone.0157232

**Published:** 2016-06-09

**Authors:** Hidehiro Umehara, Shusuke Numata, Atsushi Tajima, Akira Nishi, Masahito Nakataki, Issei Imoto, Satsuki Sumitani, Tetsuro Ohmori

**Affiliations:** 1 Department of Psychiatry, Institute of Biomedical Sciences, Tokushima University Graduate School, Tokushima, Japan; 2 Department of Bioinformatics and Genomics, Graduate School of Advanced Preventive Medical Sciences, Kanazawa University, Ishikawa, Japan; 3 Department of Human Genetics, Institute of Biomedical Sciences, Tokushima University Graduate School, Tokushima, Japan; 4 Department of support for students with special needs, Institute of Biomedical Sciences, Tokushima University Graduate School, Tokushima, Japan; Chiba University Center for Forensic Mental Health, JAPAN

## Abstract

**Background:**

Selective serotonin reuptake inhibitors (SSRI) are established first-line pharmacological treatments for obsessive-compulsive disorder (OCD), while antipsychotics are used as an augmentation strategy for SSRI in OCD patients who have either no response or a partial response to SSRI treatment. The goal of the present study was to identify genetic variants and pathways that are associated with the long-term clinical response of OCD patients to SSRI or SSRI with antipsychotics.

**Methods:**

We first performed a genome-wide association study of 96 OCD patients to examine genetic variants contributing to the response to SSRI or SSRI with antipsychotics. Subsequently, we conducted pathway-based analyses by using Improved Gene Set Enrichment Analysis for Genome-wide Association Study (i-GSEA4GWAS) to examine the combined effects of genetic variants on the clinical response in OCD.

**Results:**

While we failed to detect specific genetic variants associated with clinical responses to SSRI or to SSRI with an atypical antipsychotic at genome-wide levels of significance, we identified 8 enriched pathways for the SSRI treatment response and 5 enriched pathways for the treatment response to SSRI with an antipsychotic medication. Notably, the calcium signaling pathway was identified in both treatment responses.

**Conclusions:**

Our results provide novel insight into the molecular mechanisms underlying the variability in clinical response to SSRI and SSRI with antipsychotics in OCD patients.

## Introduction

Obsessive-compulsive disorder (OCD) is a neuropsychiatric disorder that occurs in approximately 2% of the population, and is characterized by repetitive, persistent, intrusive thoughts and repetitive, compulsive behaviors [1]. Selective serotonin reuptake inhibitors (SSRIs) are the most common first-line treatment for OCD [2], and antipsychotics have been proposed as augmenting agents in OCD patients who have either no response or a partial response to SSRI treatment. However, how to use antipsychotic drugs, such as the choice, optimal dose, and duration, has not been still established [3,4,5,6]. However, because of the variability in treatment response among OCD patients, and because several weeks are needed to reveal the efficacy of medications, the identification of biomarkers that predict treatment responses would enhance treatment outcomes in these individuals. While previous pharmacogenomics studies have focused on candidate biomarker genes, such as *BDNF* (brain-derived neurotrophic factor), *COMT* (catechol-O-methyltransferase), *CYP2D6* (cytochrome P450, family 2, subfamily D, polypeptide 6), and *SLC6A4* [solute carrier family 6 (neurotransmitter transporter), member 4], the results of these analyses have been inconsistent [7].

Genome-wide association study (GWAS) is an approach to examine the association between the particular trait and hundreds of thousands of single nucleotide polymorphisms (SNPs) across the genomes in different individuals at the same time, and have identified many genetic locus association with many complex diseases [8].

To date, only one GWAS has examined treatment responses to serotonin reuptake inhibitors (SRIs) in OCD patients [9]. Notably, this group identified single-nucleotide polymorphism (SNP), rs17162912, which is near the *DISP1* gene, associated with SRI treatment responses at genome-wide level of significance. In the current study, we aimed to further these findings by identifying genetic variants contributing to the response to SSRI or SSRI combined with antipsychotics, via GWAS analysis of 96 OCD patients.

## Materials and Methods

### Subjects

Ninety-six OCD patients were recruited from Tokushima University Hospital in Japan, as described in detail in a separate study [10]. All subjects who participated in this study were of Japanese origin, and 10 subjects had not taken any psychotropic drugs. Diagnoses of OCD were made according to the Diagnostic and Statistical Manual of Mental Disorders (DSM-IV) criteria by at least 2 expert psychiatrists. Clinical symptoms were evaluated at baseline and at the end of treatment using the Yale Brown Obsessive-Compulsive Scale (Y-BOCS). The mean follow up duration of this study was 13.1 ± 8.1 month. During the follow up period of this study, all patients had taken medications without psychotherapy treatments. After treatment, patients were divided into three groups according to their pharmacological response: group A was comprised of patients that exhibited >35% reduction in Y-BOCS scores after treatment with a high dose of SSRI (fluvoxamine 200mg-300mg, paroxetine 40mg-60mg, sertraline 150mg-200mg, or escitalopram 20mg), while group B was comprised of patients that had either no response or a partial response to SSRI treatment and exhibited a >35% reduction in Y-BOCS scores after one low-dose antipsychotic augmentation treatment (risperidone 0.25mg-3mg, olanzapine 2.5mg-10mg, quetiapine 100mg, perospirone 4mg-8mg, or aripiprazole 3mg-24mg) combined with SSRI. The remaining patients, who responded neither to SSRI treatment nor to SSRI treatment combined with antipsychotic augmentation comprised group C. The institutional ethics committee of the University of Tokushima Graduate School approved the current study and all subjects signed written, informed consent forms.

### Genotyping and Quality Control

A total of 695,789 SNPs were genotyped for each subjects using the Illumina HumanOmniExpress-24v1-0 BeadChip according to the manufacturer’s instructions(Illumina, Inc., San Diego, CA, USA). Quality control was conducted using PLINK v1.07 software. SNPs with call rates < 95%, minor allele frequencies < 5%, and Hardy-Weinberg equilibrium test P-values < 10^−6^ were excluded from the subsequent association analysis. Individuals with excessive missing genotypes, sex discrepancies, and cryptic duplicates were removed. After quality control, a total of 505,934 SNPs from 93 individuals (46 males and 47 females) were subjected to further analysis. We deposited genome-wide association study data to the Gene Expression Omnibus (GEO) of the National Center for Biotechnology Information under the accession number GSE76611.

### Statistical Analyses

To evaluate the effect of each SNP on the clinical response to SSRI (group A vs. group B plus C) or to SSRI combined with antipsychotics (group B vs. group C), logistic regression analysis was performed using PLINK v1.07 software, with adjustments for the following covariates: sex, age, onset age, and Y-BOCS baseline score. Pathway-based analyses were conducted using i-GSEA4GWAS v2 (http://gesa4gwas-v2.psycho.ac.cn/), a web server designed to enable functional analysis of SNPs in trait-associated pathways identified by GWAS [11], using the SNPs and P-values obtained from our GWAS. i-GSEA4GWAS employs SNP label permutation to correct gene variation to reduce the bias due to different genes with different number of mapped SNPs. This correction ensures to identify gene sets consisting of non-random high-association genes with biological plausibility instead of random high-association genes with large numbers of mapped SNPs [12]. This analysis has been applied in many studies [13,14,15,16,17]. The parameters used for i-GSEA4GWAS v2 analysis were as follow: SNPs were mapped to the nearest genes within their 20 kilobases (kb) upstream/downstream; the gene set/pathway databases were from KEGG (http://www/genome.jp/kegg/) and BioCarta (http://www.biocarta.com); and only gene sets/pathways comprising at least 20 and at most 200 genes were examined. A false discovery rate (FDR) correction of 0.05 was applied for multiple testing. The pathways/gene sets with FDR < 0.25 is regarded as ‘possible`or `hypothesis`, while the threshold of FDR < 0.05 is regarded as ‘high confidence`or ‘with statistical significance`in this analysis [12].

## Results

### Demographics

After quality control, a total of 93 patients were divided into three groups according to their pharmacological responses. Specifically, groups A, B, and C were comprised of, 56, 23, and 17 patients, respectively. The clinical characteristics of the patients in each group are summarized in Table 1. No significant differences in gender, age, onset age, or duration of illness was observed among the three groups. However, significant differences were observed among the groups in Y-BOCS scores at baseline and at the end of the treatment.

**Table 1:** Treatment response group and clinical symptoms of patients with obsessive-compulsive disorder.

	A	B	C		
	(N = 54)	(N = 22)	(N = 17)	total	Pvalue
Sex (M), N (%)	24 (44.4)	12 (54.5)	10 (58.8)	46	0.51
Sex (F), N (%)	30 (55.6)	10 (45.5)	7 (41.2)	47	
Age, mean (SD)	33.28 (12.84)	30.41 (11.33)	28.41 (9.86)	31.71(11.98)	0.3
Onset age, mean (SD)	25.07 (10.77)	23.14 (12.17)	18.00 (10.88)	23.32(11.26)	0.079
Duration of illness, mean (SD)	8.20 (9.29)	7.27 (6.17)	10.41 (10.03)	8.39(8.72)	0.53
Y-BOCS score					
Baseline, mean (SD)	23.57 (4.85)	25.55 (4.62)	28.00 (4.30)	24.85(4.93)	0.003
After treatment, mean (SD)	8.28 (3.89)	11.36 (3.02)	25.12 (4.82)	12.08(7.37)	<0.001
P-values are calculated by χ2 and ANOVA.					
Duration of illness: length from onset of obsessive complusive disorder to start of treatment in Tokushima university hospital.					
A: Responders to a selective serotonin reuptake inhibitor					
B: Responders to a selective serotonin reuptake inhibitor with an atypical antipsychotic					
C: Non-responders to a selective serotonin reuptake inhibitor with an atypical antipsychotic					

### Genome-wide Association Study

After quality control, a total of 505,934 SNPs were examined in the present pharmacogenomics study. Of these, 178 were nominally associated with the SSRI treatment response, and one was nominally associated with the treatment response to SSRI in combination with an antipsychotic medication (P < 1.0 × 10^−3^). However, none of these SNPs exhibited genome-wide levels of significance (P < 5 × 10^−8^). The 10 SNPs exhibiting the strongest associations with each treatment response are shown Table 2, and a list of SNPs with P values < 1.0 x 10^−3^ is included in supplement file (S1 Table). Notably, rs6557479, which is located near the *ARID1B* (AT-rich interactive domain 1B) gene, was among the 10 SNPs with the strongest associations with SSRI treatment response. ARID1B encodes a DNA binding subunit of the Brahma-associated factor chromatin remodeling complex, which plays a key role in the regulation of gene activity and neurodevelopment [18], and mutations in this gene have been associated with autism, intellectual disability, and developmental delay [19,20,21].

**Table 2:** Association results of GWAS for the treatment response in OCD.

**SSRI treatment response**								
SNP	Chr	Position	Alleles	MAF	OR	P	Genomic Location	Closest gene
rs6118017	20	7924726	[A/G]	0.4177	5.732	6.26×10–5	Intergenic	HAO1
rs6557479	6	156502670	[T/C]	0.375	7.023	1.03×10–4	Intergenic	ARID1B
rs11778051	8	141410497	[T/C]	0.4415	4.511	1.35×10–4	Intronic	TRAPPC9
rs2876110	20	7947638	[A/G]	0.4266	4.897	1.39×10–4	Intergenic	HAO1
rs10882614	10	97315080	[T/C]	0.1468	0.06546	1.41×10–4	Intronic	SORBS1
rs2474028	14	92403660	[T/C]	0.2272	0.1973	1.46×10–4	Intronic	FBLN5
rs7241999	18	31640468	[T/C]	0.4692	0.2053	1.57×10–4	Intronic	NOL4
rs1543377	20	7951007	[T/G]	0.4216	4.773	1.87×10–4	Intergenic	HAO1
rs7599124	2	174455978	[A/G]	0.2242	0.149	2.06×10–4	Intergenic	CDCA7
rs1963569	4	169338520	[A/C]	0.253	0.2539	2.18×10–4	Intronic	DDX60L
**The treatment response to SSRI with an antipsychotic medication**								
SNP	Chr	Position	Alleles	MAF	OR	P	Genomic Location	Closest gene
rs3812398	7	29237994	[A/G]	0.2837	0.01382	8.92×10–4	Intronic	CHN2
rs2004915	11	12127181	[T/C]	0.3363	0.04343	1.11×10–3	Intronic	MICAL2
rs2838416	21	45252838	[T/C]	0.1954	0.0303	1.21×10–3	Intergenic	AGPAT3
rs11914777	3	32334008	[T/C]	0.2004	0.04424	1.21×10–3	Intronic	CMTM8
rs7955451	12	100005790	[T/G]	0.4683	0.07164	1.31×10–3	Intronic	ANKS1B
rs1039378	12	99991075	[T/C]	0.4812	0.0374	1.32×10–3	Intronic	ANKS1B
rs12151194	19	53552296	[A/C]	0.3313	0.04769	1.36×10–3	Intronic	ERVV-2
rs6509718	19	53548950	[A/G]	0.3165	0.04769	1.36×10–3	Intronic	ERVV-2
rs8128681	21	47049185	[T/C]	0.1567	0.04903	1.50×10–3	Intergenic	PCBP3
rs6133566	20	8254666	[T/C]	0.3452	38.13	1.55×10–3	Intronic	PLCB1

Chr, chromosome; MAF, minor allele frequency; NA, not applicable; OR, odds ratio; SNP, single-nucleotide polymorphism.

Meanwhile, the SNP that exhibited the strongest association with the response to SSRI combination with antipsychotic medication was rs3812398, which is located in the *CHN2* (chimerin 2; P = 8.9 × 10^−4^) gene. CHN2, which is expressed in a variety of human tissues but is expressed at the highest levels in the brain, is GTPase-activating protein that plays an important role in the establishment of functional brain circuitry by controlling axon pruning [22]. Mutations in this gene have been associated with schizophrenia and atypical psychosis [23,24].

### Pathway Analysis

Table 3 lists the significantly enriched pathways for each treatment response. Eight pathways were identified from the results of the GWAS for the SSRI treatment response, while 5 pathways were identified from the results of the GWAS for the treatment response to SSRI in combination with an antipsychotic medication (FDR values < 0.05). Among these pathways, the calcium signaling pathway was significantly enriched in both treatment responses.

**Table 3:** Pathway-based analysis for the results of GWAS for the treatment response in OCD.

**SSRI treatment response**		
Gene Set Name	P-value	FDR value
Axon guidance (KEGG)	0.001	0.0125
FCER1 signaling pathway (BioCarta)	0.001	0.0128
Fc epsilon RI signaling pathway (KEGG)	0.001	0.0130
T cell receptor signaling pathway (KEGG)	0.001	0.0137
TCR pathway (BioCarta)	0.002	0.0438
Calcium signaling pathway (KEGG)	0.002	0.0459
NFAT pathway (BioCarta)	0.003	0.0489
GPCR pathway (BioCarta)	0.002	0.0497
**The treatment response to SSRI with an antipsychotic medication**		
Gene Set Name	P-value	FDR value
Calcium signaling pathway (KEGG)	0.001	0.0070
Dilated cardiomyopathy (KEGG)	0.001	0.0345
Nitrogen metabolism (KEGG)	0.003	0.0347
mTOR pathway (BioCarta)	0.001	0.0365
Long-term potentiation (KEGG)	0.002	0.0418
Pathways with nominal P≤0.01 and FDR<0.05		

## Discussion

While we identified suggestive 178 SNPs that had significant associations with long-term clinical responses to SSRI in OCD patients, none of the associations exhibited the genome-wide levels of significance. To reveal combined effects of SNPs on SSRI response, we conducted a pathway-based analysis using our GWAS data and identified eight pathways associated with treatment responses. Of these, the FCER1 pathway, the Fc epsilon RI signaling pathway, the T cell receptor (TCR) signaling pathway, and the TCR pathway were associated with immune system function. Consistent with our findings, previous studies have linked immune system dysfunctions, such as alterations in cytokine productione, with OCD [25,26]. Moreover, SSRIs have been shown to exert immunological effects, such as reduced lymphocyte proliferation, alterations in cytokine secretion, and induction of apoptosis [27]. The axon guidance exhibited the strongest association with the response to SSRI treatment in OCD patients. In previous studies, microRNA target genes have been enriched in several pathways during SSRI treatment, including axon guidance [28]. The calcium signaling pathway was also exhibited significant association with the response to SSRI treatment in OCD patients. Ca^2+^ signaling is an important intracellular signal that modulates many different cellular functions, including the central nervous system [29]. SSRIs affect Ca^2+^ signaling through the regulation of intracellular Ca^2+^ concentrations, and thereby modulate cell proliferation, immune system function, and gene transcription, as well as exocytosis at synapses in several cell types, including neurons, astrocytes, microglia, platelets, and lymphocytes [30,31,32].

To date, one GWAS has examined treatment responses to SRIs in OCD patients [9]. However, none of the 42 SNPs identified by Qin and colleagues that exhibited significant associations (P < 10−^4^) with SRI responses in OCD patients were among the 178 SNPs that exhibited nominal associations with SSRI response (P < 10^−3^) in the present study. These inconsistent results between studies might be due to differences in sample size (93 vs. 804), antidepressant drug treatment (SSRI vs. SRI, including serotonin and norepinephrine reuptake inhibitor, and a tricyclic antidepressant), dosages used for treatment, treatment duration (13.1 ± 8.1 months vs. lack of detailed information), and/or to differences in the methods used for evaluation and definition of treatment responses (Y-BOCS scores vs. self-report).

To the best of our knowledge, this is the first GWAS to examine genetic variants contributing to the response of OCD patients to SSRI combined with antipsychotic therapy. Here, we identified one suggestive SNP that showed a significant association with patient responses to such treatment; however, the link between this SNP and patient outcomes did not reach the genome-wide level of significance. We subsequently detected five pathways that were significantly enriched in this treatment group. Of these, the calcium signaling pathway exhibited the strongest association with patient responses to SSRI combined with antipsychotic therapy. Notably, this pathway was also associated with the response to SSRI treatment in OCD patients. Consistent with these findings, previous studies have suggested that antipsychotics target microglial intracellular Ca^2+^ signaling [33], and that the combined SSRI-antipsychotic treatment exerts selective effects on components of the calcium cascade in prefrontal cortex of rat and human peripheral mononuclear cells [34,35].

There are several limitations to the present study. First, the sample size was limited to enable the detection of small effects of genetic variants on treatment responses, and we failed to detect specific genetic variants associated with clinical responses at genome-wide levels of significance (P < 5 × 10^−8^) although we revealed several enriched pathways (FDR < 0.05). Further studies using a large cohort will therefore be necessary to confirm our findings. Second, the patients that comprised our cohort were treated in a naturalistic clinical course, and were treated with a variety of medications. As such, studies using patients who are taking a single antidepressant are needed. Finally, clinical characteristics of the patients in each pharmacological response group might affect our results although logistic regression analysis was performed to examine the effect of SNP on the clinical response with adjustments for sex, age, onset age, and Y-BOCS baseline score.

In conclusion, we have identified several pathways that may contribute to the response of OCD patients to SSRI and/or SSRI combined with antipsychotic treatments. Of these, several were associated with cellular mechanisms known to be affected by SSRIs or antipsychotics. Our results provide novel insight into the molecular mechanisms underlying clinical response variability to SSRI and SSRI with antipsychotics in OCD patients.

## Supporting Information

S1 Table Association results of GWAS for the SSRI treatment response in OCD (P values < 1.0 x 10^−3^). (XLSX)
